# Comparison of the global gene expression of choroid plexus and meninges and associated vasculature under control conditions and after pronounced hyperthermia or amphetamine toxicity

**DOI:** 10.1186/1471-2164-14-147

**Published:** 2013-03-05

**Authors:** John F Bowyer, Tucker A Patterson, Upasana T Saini, Joseph P Hanig, Monzy Thomas, Luísa Camacho, Nysia I George, James J Chen

**Affiliations:** 1 Division of Neurotoxicology, National Center for Toxicological Research, U.S. Food and Drug Administration, Jefferson, AR 72079-9502, USA; 2 Division of Biochemical Toxicology, National Center for Toxicological Research, U.S. Food and Drug Administration, Jefferson, AR, 72079-9502, USA; 3 Division of Bioinformatics and Biostatistics, National Center for Toxicological Research, U.S. Food and Drug Administration, Jefferson, AR, 72079-9502, USA; 4 Center for Drug Evaluation and Research, U.S. Food & Drug Administration, Silver Spring, MD, 20993, USA

**Keywords:** Gene expression, Meninges, Cerebral vasculature, Choroid plexus, Cerebrospinal fluid, Amphetamines, Hyperthermia

## Abstract

**Background:**

The meninges (arachnoid and pial membranes) and associated vasculature (MAV) and choroid plexus are important in maintaining cerebrospinal fluid (CSF) generation and flow. MAV vasculature was previously observed to be adversely affected by environmentally-induced hyperthermia (EIH) and more so by a neurotoxic amphetamine (AMPH) exposure. Herein, microarray and RT-PCR analysis was used to compare the gene expression profiles between choroid plexus and MAV under control conditions and at 3 hours and 1 day after EIH or AMPH exposure. Since AMPH and EIH are so disruptive to vasculature, genes related to vasculature integrity and function were of interest.

**Results:**

Our data shows that, under control conditions, many of the genes with relatively high expression in both the MAV and choroid plexus are also abundant in many epithelial tissues. These genes function in transport of water, ions, and solutes, and likely play a role in CSF regulation. Most genes that help form the blood–brain barrier (BBB) and tight junctions were also highly expressed in MAV but not in choroid plexus. In MAV, exposure to EIH and more so to AMPH decreased the expression of BBB-related genes such as *Sox18, Ocln,* and *Cldn5*, but they were much less affected in the choroid plexus. There was a correlation between the genes related to reactive oxidative stress and damage that were significantly altered in the MAV and choroid plexus after either EIH or AMPH. However, AMPH (at 3 hr) significantly affected about 5 times as many genes as EIH in the MAV, while in the choroid plexus EIH affected more genes than AMPH. Several unique genes that are not specifically related to vascular damage increased to a much greater extent after AMPH compared to EIH in the MAV *(Lbp, Reg3a, Reg3b, Slc15a1, Sct* and *Fst*) and choroid plexus (*Bmp4*, *Dio2* and *Lbp*).

**Conclusions:**

Our study indicates that the disruption of choroid plexus function and damage produced by AMPH and EIH is significant, but the changes may not be as pronounced as they are in the MAV, particularly for AMPH. Expression profiles in the MAV and choroid plexus differed to some extent and differences were not restricted to vascular related genes.

## Background

The choroid plexus and the meninges (arachnoid and pial membranes) and associated vasculature (MAV) form an entity that is of utmost importance in regulating brain blood flow and cerebrospinal fluid (CSF) integrity. As well, they are critical in maintaining the neuronal integrity of the brain, particularly the cerebral hemispheres [1-10]. This is the case when damage to vasculature within specific brain regions in the cerebral hemispheres occurs from neurotoxic doses of amphetamine (AMPH) in laboratory animals [11-14] and the abuse or illegal use of AMPH and methamphetamine (METH) in humans [15-19]. Unlike other well characterized cerebrovascular insults [6,8,20-23], much less is known about how amphetamines compromise the vasculature in MAV, the choroid plexus, and areas within the cerebral hemispheres. Our previous studies [24,25] examined how amphetamines adversely affect MAV vasculature in laboratory animals. However, the effects of neurotoxic doses of amphetamine on choroid plexus function have not been previously addressed.

In the MAV, AMPH and METH directly release norepinephrine from the terminals of adrenergic input to the arteries and arterioles in the pial layer causing vasoconstriction and bypassing neuronal impulse regulation of release from the sympathetic ganglia, which normally auto-regulates cerebral blood flow (CBF) [2-4]. Although this may initially be responsible for reduced CBF in laboratory animals, it is not the only cause [13]. In a previous study, we observed increased changes in the expression of genes related to regulating CBF, including endothelin1 (*Edn1)* and nitric oxide synthase (*Nos1)*, that may be initiated to overcome the AMPH dysregulation of CBF; the lack of increased vasointestinal peptide (*Vip*) may be a deficiency induced by AMPH [24]. Not only does neurotoxic AMPH exposure cause a prominent alteration in regulation of vascular tone, it also disrupts the expression of genes related to angiogenesis and produces a significant endoplasmic reticulum stress response (ERSR) [24,25]. As of yet, it is not specifically known how neurotoxic exposures to AMPH and METH affect choroid plexus vasculature and CSF production.

In the current work, efforts were taken to identify genes in MAV, as well as the choroid plexus, that are responsible for regulating CSF composition and flow because alteration in CSF function could lead to significant adverse effects on brain function [6,8]. Recent laboratory animal research indicates that most CSF exits the brain through the lymphatic system around the cranial nerves rather than the arachnoid villi residing at the most dorsal aspect of the meninges [5,26-28]. This suggests a CSF flow more dorsally directed, which could well necessitate the MAV playing a more prominent role in regulating CSF flow and composition. As a means of trying to identify genes involved in the regulation of CSF flow and composition, we first compared the global gene expression profiles under control conditions in the neuronal-rich tissues striatum and parietal cortex to those in the vascular-rich tissues choroid plexus and MAV. This enabled the identification of genes with 15-fold or greater expression in MAV and choroid plexus compared to striatum and parietal cortex. In addition to identifying genes related to CSF composition and flow, our study further aimed at identifying genes with much higher expression in MAV and choroid plexus than in striatum and parietal cortex that could be of great importance in other specific functions (not CSF) of MAV and choroid plexus and possibly more likely to be more significantly affected by AMPH.

Additionally, we examined gene expression changes resulting from environmentally-induced hyperthermia (EIH), since the neurotoxicity [29-33] and vascular effects [11,12,14,31] of AMPH and METH are highly temperature-dependent. Importantly, hyperthermia alone adversely affects the vasculature of the brain [34-37]. As well, EIH toxicity in rats has many similar characteristics to heat stroke in humans, which has been reported to have neurotoxic and vascular effects [34-36,38]. However, unlike AMPH and METH, EIH does not produce dopamine terminal damage or diffuse neurodegeneration in the parietal cortex, limbic cortex, and thalamus. Also, EIH does not arrest angiogenesis or elevate endothelial nitric oxide synthase1 (*Nos1*) in MAV as does AMPH [24,25]. As with neurotoxic AMPH and METH exposures, little is known regarding the effects of EIH on choroid plexus vasculature and CSF production.

Through the use of oligo array and RT-PCR technologies, the present study evaluates global gene expression changes resulting from the adverse and damaging effects of neurotoxic exposures of AMPH and EIH in rat mRNA from the MAV and choroid plexus. Gene expressions were measured from RNA generated from tissues obtained from our previous studies [24,25] and from additional new RNA samples obtained from the choroid plexus and MAV. Particular interest was focused on comparing fold-changes of genes related to vascular tight junctions and the blood–brain barrier (BBB) in MAV and choroid plexus because a loss of integrity of tight junctions in these two structures would likely lead to significant adverse effects. One of the main goals of the study is to identify genes in MAV and choroid plexus that are not necessarily known to affect vascular function and genes that are not well characterized.

## Results

In the current study, we present the effects of AMPH and EIH on global gene expression levels in the choroid plexus via array analysis (found at: GEO, NCBI; data file GSE29733) and compare these effects with those induced in the MAV, striatum, and parietal cortex (GEO, NCBI; data file GSE23093) on a genome-wide scale. The expression profile of selected genes was further evaluated/confirmed using RT-PCR for all four tissues/brain regions. Changes in body temperature and behavior have previously been reported for control, EIH, and AMPH exposed animals [24,25]. The additional rats dosed for obtaining more choroid plexus and MAV tissue samples had the same temperature profiles as those previously exposed to AMPH and EIH (data not shown). Array and RT-PCR data for the choroid plexus have not been reported previously nor has the global gene expression in choroid plexus ever been compared to that in MAV, striatum, or parietal cortex. Changes in MAV, striatum, or parietal cortex expression after either EIH or AMPH exposure for some genes known to regulate vascular tone, angiogenesis, and endoplasmic reticulum stress response (ERSR) have been reported in the two aforementioned manuscripts. As well, a manuscript/video presentation [39], which shows dissection methods for the MAV, contains a table showing the large fold-differences in MAV gene expression with expression in striatum and cortex. However, complete analysis and comparison of the global gene expression in MAV under control, EIH, or AMPH conditions have never been presented, and MAV gene expression has not been previously compared with choroid plexus.

### Control expression profiles in choroid plexus, MAV, striatum and parietal cortex

#### Global gene expression under control conditions

Gene expression data in the control animals were used to generate Additional file 1: Table S1, which presents summary statistics (mean and standard deviation) of gene expression data (>11,000 genes) from the MAV, striatum, parietal cortex, and choroid plexus. Table 1 shows genes in the choroid plexus that have a 15-fold or greater expression in choroid plexus than in striatum and parietal cortex under control conditions. Genes in MAV that have a 15-fold higher expression than the striatum and parietal cortex can be found in [39] and Additional file 2: Table S2. Genes with a 15-fold or higher expression level in MAV and choroid plexus, relative to the striatum and parietal cortex (neuronal-rich tissues), were identified as being genes of particular importance to the function of these neuronal-sparse tissues. Genes with higher expression (5-fold or more) in circulating blood (GSE29733; present in leukocytes or reticulocytes) than in the MAV and choroid plexus were not included in this list to exclude the possibility that the high expression levels in the MAV and choroid plexus were due to residual blood in these tissues. There was a significant number of genes with 15-fold or higher expression in the MAV and the choroid plexus compared to the striatum and parietal cortex that were directly related to the vascular system and/or the immune system, but they were not in the majority (Table 1 and Additional file 2: Table S2). A number of the genes with very large fold changes were: 1) extracellular matrix proteins (not specifically related to vasculature), 2) solute transporters, and 3) proteins involved in lipid and retinoic acid metabolism. As well, a few growth and differentiation genes or transcription factors were found. The overlap in the genes with a 15-fold or greater expression in MAV and choroid plexus relative to neuronal tissues was over 50% (60% for 10-fold or greater genes) (Table 1).

**Table 1:** Putative function of genes with increased expression of 15-fold* or more in choroid plexus compared to striatum and parietal cortex under control conditions

**Choroid plexus expression relative to striatum and parietal cortex expression**	**NCBI gene symbols**	**Tissue specificity or reported function(s)**
> 50-fold	***Col8a1, Esm1, Glycam1, Lect1, Thbs2***	Vasculature & Heart
> 50-fold	*A2m, ****Pla2g5, Xcl1***	Immune System
> 50-fold	*Adipoq, Pon1, ****Pon3***	Retinoic Acid & Lipid Processing
> 50-fold	*Cldn1, ****Crb3, Fmod, Kl***^***a***^***, Steap1, Tmem27***	Extracellular Matrix & Cell-Cell Junctions
> 50-fold	***Aqp1****, Atp4a, Clic6,Kcne2, ****Kcnj13, Slc4a5, Slc5a5****, Slc16a8, ****Slco1a5, Ttr****, Trpm3*	Ion & Solute Transport & Homeostasis
> 50-fold	***Cdkn1c, Folr1, Igfbp2, Mpzl2, Msx1,Otx2, Sostdc1***	Development & Transcription Regulation
> 50-fold	*Cox8h, Efhb, ****Epn3****, Fscn2, ****Klc3, Krt18, Scgb1c1***	Unknown & Miscellaneous
30 to 50-fold	***Lepr, Mdk, Ogn****, Tnni2*	Vasculature & Heart
30 to 50-fold	***Cd74***^***a***^***, Klra5****, Nphs1, ****RT1-Da, Serpinb1a***	Immune System
30 to 50-fold	*Cldn2, ****Dcn, Lama2, Lgals3bp****, Rsph4a*	Extracellular Matrix & Cell-Cell Junctions
30 to 50-fold	*Slc4a2,Slc26a7*	Ion & Solute Transport & Homeostasis
30 to 50-fold	*Apoa2, Lrat*	Retinoic Acid & Lipid Processing
30 to 50-fold	*Msx3, NTF4, ****Wfikkn2***	Development & Transcription Regulation
30 to 50-fold	*Atp11c, ****Copz2, Dhrs7c****, Dmrt3,F5, Klk4,Krt8, Nt5dc2, ****Pdlim2****, ****Phactr2****, Sah, Scara5, Spag8,*	Unknown & Miscellaneous
15 to 30-fold	*Ace****, Anxa2***^***a***^***, Cox8h****, Itgb6, ****Trim63***	Vasculature & Heart
15 to 30-fold	*Cmtm8, ****Ctsc****, Igsf1, ****RT1-Db1***	Immune System
15 to 30-fold	***Adamtsl4****, Col9a3, Dsp, ****Loxl1, Mmp14****,Prelp, Sulf1*	Extracellular Matrix & Cell-Cell Junctions
15 to 30-fold	*Fxyd1, Slc12a4,Slc16a12,Slc19a1, Slc25a30, ****Sned1***	Ion & Solute Transport & Homeostasis
15 to 30-fold	***Agpat2, Enpp2, Gprc5c****, Rbp2*	Retinoic Acid & Lipid Processing
15 to 30-fold	*Cdkn1a, ****Dab2, Igf2****, Nr1i2, ****Prrx2, Sfrp1, Zic4***	Development & Transcription Regulation
15 to 30-fold	*Akap14, Capn3, Capsl, Ccdc37, ****Dse****, Elmo3, Hdc, ****Hspb1****, Iqcg, Kpl2, Lhb, ****Mesp1****, Nudt7, Otc, Paqr5, ****Ppp1r3b, Pqlc3****, Tsga2, Sod3, Spata6,Spint2, Sult1c1*	Unknown & Miscellaneous

* Genes included in the table must have been more than 15-fold above expression in the choroid plexus compared to both the striatum and parietal cortex and at least 5-fold above the background level. Also, expression must have been relatively consistent across individual control animals so that the S.D. was less than the mean. The lower of the two ratios (choroid plexus/striatum or choroid plexus/parietal cortex) for each gene was used for grouping. Genes were categorized using *NCBI Entrez Gene.*

^a^Genes found in endothelial cells that also likely play a major role in mediating immune responses.

Bold font indicates gene expression was also at least 15-fold greater in MAV compared to both the striatum and parietal cortex.

Underlined font indicates gene expression was also at least 10- to 15-fold greater in MAV compared to both the striatum and parietal cortex.

#### Levels of vasculature-related genes in choroid plexus, MAV, striatum, and cortex

The mRNA expression levels of genes normally ascribed to be present in almost all types of vascular endothelium (not just BBB or tight junction related), such as intercellular adhesion molecule 1 *(Icam1),* kinase insert domain receptor *(Kdr/Vegfr2),* nitric oxide synthase 3 *(Nos3), and* vascular cell adhesion molecule 1 *(Vcam1)* were 5- to 10-fold greater in MAV than in the parietal cortex and striatum under control conditions (Table 2). These higher levels suggest that there is likely a 5- to 10-fold greater vascular content in the MAV compared to striatum and parietal cortex. In contrast, in the choroid plexus most of the vascular associated genes that were not linked to tight junctions or the BBB were at the same level as the neuronal tissues, with the exception of *Icam1* and *Kdr* levels which were in the MAV range. This was also true for most of the genes related to angiogenesis (*e.g*., *Ctgf, Timp1 Vegfc, Anpep, Col18a1*); only *Mmp2* and *Akt1* were at or above MAV levels in the choroid plexus (Table 2). In contrast, genes related to the ERSR pathways were expressed at very similar levels in the MAV and choroid plexus (Additional file 1: Table S1).

**Table 2:** Normalized expression of selected genes in control animals for all four brain regions determined by oligo array analysis

	**Brain region**							
	**MAV**		**Parietal cortex**		**Striatum**		**Choroid plexus**	
***NCBI gene***	**3 hr control**		**3 hr control**		**3 hr control**		**3 hr control**	
***Symbol***	**Mean ± S.D.**		**Mean ± S.D.**		**Mean ± S.D.**		**Mean ± S.D.**	
*Anpep*	441.7	57.8	52.9	11.4	34.6	9.3	83.0	12.9
*Akt1*	666.1	62.8	1185.9	88.1	1164.0	169.1	983.6	88.1
*Aqp1*	2433.0	721.3	33.9	2.6	31.0	5.4	8576.6	513.3
*Aqp4*	70.0	27.4	42.2	6.5	169.7	39.6	35.7	24.6
*Cldn1*	446.5	305.9	8.6	3.8	7.5	2.1	2075.5	235.0
*Cldn2*	46.9	40.5	6.3	3.2	7.0	3.5	350.6	55.1
*Cldn3*	68.0	13.0	127.9	29.5	82.4	10.7	327.1	4.7
*Cldn5*	9721.0	2380.3	2222.5	784.9	2505.1	461.6	274.4	74.7
*Cldn9*	2248.0	380.2	147.3	34.4	364.0	86.0	1335.8	121.8
*Col18a1*	1523.7	335.7	48.4	16.1	183.0	33.1	148.9	44.7
*Ctgf*	4842.1	1174.6	666.7	190.5	540.2	188.6	224.6	47.2
*Icam1*	1186.5	151.9	101.3	12.1	84.9	8.1	508.5	18.2
*F11r*	1134.3	205.4	176.3	5.9	167.3	20.5	1602.3	200.7
*Jam3*	418.9	113.0	295.4	34.3	357.6	53.9	1318.4	94.0
*Kdr*	12737.6	1911.1	1310.9	319.1	1604.2	272.0	6323.5	874.5
*Mmp2*	1003.8	609.1	96.7	18.7	105.5	21.7	7657.0	986.9
*Mmp14*	5519.5	1293.1	195.9	86.8	277.3	77.2	6744.9	1065.3
*Nos3*	757.9	157.5	174.6	27.1	145.5	14.6	91.4	25.4
*Ocln*	1611.4	291.8	183.3	26.2	253.5	13.1	3503.3	311.4
*Timp1*	7226.9	1483.0	260.3	50.7	224.2	45.3	888.1	88.7
*Tjp2*	337.2	60.5	291.6	18.9	336.5	16.9	286.4	49.5
*Tjp3*	183.4	138.9	39.5	7.3	97.9	5.7	1427.6	217.9
*Vcam1*	12434.4	2077.7	2604.2	222.6	3787.3	356.9	2660.5	195.6
*Vegfc*	1750.4	333.1	658.7	129.1	310.4	29.8	548.1	33.5

Under control conditions, the expression of many genes associated with tight junctions or BBB that are present in the vasculature of neuronal-rich tissues, such as the parietal cortex and striatum, were as high in MAV even if it is assumed that the vasculature in MAV was 5- to 10-fold greater (previous paragraph). However, the “relative” expression levels of claudin 3 (*Cldn3*), tight junction protein 2 (*Tjp2*), and aquaporin 4 (*Aqp4*) in MAV may be many-fold below that of parietal cortex and striatum due to MAV having such a greater proportion of vasculature. The expression of solute carrier family 9 (sodium/hydrogen exchanger, *Slc9a2*), a gene implicated in tight junctions [40], is higher than expected in MAV. The choroid plexus expressed high levels of some genes that are involved in tight junctions such as *Ocln, Cldn1, Cldn2, Cldn9, F11r, Jam3,* and *Tpj3,* but expression profiles of the various tight junction genes in this tissue differed somewhat from MAV and even more so from striatum and parietal cortex (Table 2).

### Effects of AMPH and EIH on gene expression

#### Genes related to vascular tone, ERSR, angiogenesis, and tight junctions

In the choroid plexus, the effects of EIH and AMPH on many genes related to vascular tone (*e.g., Edn1, Nos3, and Vip*) and angiogenesis did not reach significance, with the exception of *Adora1a* and *Adora1b* (Table 3). In contrast, in the MAV, EIH and/or AMPH significantly increased *Edn1, Nos3,* and *Vip* levels but not *Adora1a* and *Adora1b* ([24], Additional file 3: Table S3). Also, more BBB genes were differentially affected in MAV compared to choroid plexus after EIH and AMPH exposure within 3 hr. *Sox18*, *Ocln, Cldn5*, and *Jam2* expressions were prominently decreased by EIH and/or AMPH (in most cases to a greater extent by AMPH) in MAV (Table 4), as well as in the striatum and parietal cortex (data not shown). However, none of these four genes were significantly affected in the choroid plexus (Table 4). Array data indicated that *Esam* was increased by AMPH in the MAV, striatum, and parietal cortex, while in the choroid plexus both AMPH and EIH tended to increase *Esam* expression (Table 4). RT-PCR analysis confirmed the significant *Esam* increases in the striatum and cortex but not in the MAV (Table 5). Array data analysis indicated that *Tjp1*, *Tjp2,* and *Tjp3* were not affected in choroid plexus or MAV by EIH or AMPH (Table 3 and Additional file 3: Table S3).

**Table 3:** Genes in choroid plexus significantly affected 3 hr after either AMPH or EIH compared to control group (FDR = 0.10)

**AMPH group expression relative to control**		**EIH group expression relative to control**	
NCBI Gene Symbol	Fold-Change	NCBI Gene Symbol	Fold-Change
*Abcc8*	3.39	*Abcc8*	1.79
*Acat1*	0.59	*Abhd2_predicted*	1.47
*Adora2b*	3.21	*Adora2a*	2.75
*Ahsa2_predicted*	5.27	*Adora2b*	3.7
*Arg2*	6.4	*Ahsa2_predicted*	6.6
*Arid5a*	3	*Ak2*	1.68
*Atf3*	7.69	*Angptl4*	2.27
*Bag3*	6.5	*Ap1s2_predicted*	2.03
*Bmp4*	4.52	*Arg2*	7.99
*C1qr1*	3.03	*Arhgef5*	1.65
*Cables1_predicted*	2.67	*Arid5a*	2.97
*Cacybp*	2.58	*Armet_predicted*	2.64
*Cd14*	2.05	*Atf3*	13.16
*Cebpd*	9.67	*Atg16l1_predicted*	2.1
*Chordc1_predicted*	2.63	*Azi2*	1.83
*Ctgf*	4.06	*Bag3*	12.93
*Cxcl1*	60.84	*Bcl6_predicted*	2.4
*Cyr61*	9.73	*Bicd2*	1.75
*Dusp1*	6.32	*Birc3*	2.49
*Emr1*	0.53	*Bst2*	2.7
*Fkbp4*	3.32	*Btg1*	1.84
*Flrt2_predicted*	2.67	*Btg2*	2.4
*Fos*	28.1	*C1qr1*	3
*G0s2*	0.29	*Cables1_predicted*	2.05
*Gata1*	4.86	*Cacybp*	2.72
*Gda*	3.26	*Calr*	1.63
*Gjb2*	0.19	*Carhsp1*	1.45
*Has1*	11.3	*Cars_predicted*	1.47
*Hmox1*	7.19	*Cblb*	1.6
*Hspa1a*	56.38	*Ccl2*	6.64
*Hspa4l_predicted*	2.74	*Ccl7*	2.28
*Hspb1*	12.83	*Cd14*	2.36
*Hspca*	3.23	*Cd24*	1.74
*Hspcb*	2.08	*Cdx4_predicted*	1.92
*Hspd1*	3.51	*Cebpb*	4.24
*Hspe1*	2.54	*Cebpd*	8.85
*Hsph1*	5.58	*Cflar*	3.01
*Icam1*	2.33	*Chac1_predicted*	2.86
*Id4*	3.21	*Chordc1_predicted*	2.69
*Il1b*	3.69	*Creld2*	2.72
*Il1r2*	9.89	*Crem*	1.82
*Il1rn*	4.15	*Cryab*	3.4
*Il6st*	2	*Ctgf*	4.44
*Inhbb*	8.3	*Cxcl1*	55.72
*Junb*	6.14	*Cxcl2*	24.79
*Litaf*	2.29	*Cxcr4*	2.29
*Lman2l_predicted*	2.56	*Cyr61*	13.53
*Nfkbia*	2.24	*Dapk3*	1.56
*Nr4a3*	16.67	*Ddit3*	2.57
*Ntel1*	1.6	*Ddx18*	1.44
*P4ha1*	4.11	*Ddx50*	1.72
*Phlda1*	2.22	*Dlc1*	2
		*Dnaja1*	2.26
*Pla2g1b*	3.48	*Dnaja4*	2.45
*Plagl1*	3.67	*Dnajb1_predicted*	4.68
*Plod1*	1.84	*Dnajb11*	1.85
*Polg2_predicted*	1.84	*Dnajb4*	2.39
*Pspla1*	2.47	*Dnajb9*	2.1
*Rgs1*	11.34	*Dnajc3*	1.51
*Sectm1b*	13.19	*Dusp1*	5.04
*Serpine1*	10.33	*Dusp11*	1.82
*Serpinh1*	2.56	*Egr1*	4.39
*Slc5a3*	2.06	*Errfi1*	3.75
*Slco1c1*	1.63	*Ets2*	1.81
*Slpi*	8.21	*Exosc3_predicted*	2
*Sphk1*	3.32	*Fbln1_predicted*	2.11
*Spry1_predicted*	4.04	*Fbxl3*	1.56
*Stat3*	1.83	*Fbxo30*	1.87
*Stip1*	2.62	*Fgl2*	5.87
*Timp1*	3.15	*Fkbp4*	3.77
*Tmem63c_predicted*	2.6	*Flrt2_predicted*	3.72
*Tnfrsf12a*	4.32	*Fos*	8.5
*Trib3*	2.82	*Gadd45b*	4.2
*Verge*	10.57	*Gda*	2.52
		*Gja7*	2.41
		*Gls*	1.78
		*Gpnmb*	1.5
		*Gpr4*	2.42
		*Gprasp1*	1.68
		*Hbegf*	2.61
		*Herpud1*	1.87
		*Hist1h2bl*	1.52
		*Hmox1*	19.07
		*Hnrpc*	1.73
		*Hspa1a*	115.67
		*Hspa4l_predicted*	2.1
		*Hspb1*	15.27
		*Hspb8*	2.2
		*Hspca*	3.4
		*Hspcb*	2.32
		*Hspd1*	4.53
		*Hspe1*	2.9
		*Hsph1*	7.2
		*Hyou1*	2.83
		*Icam1*	2.46
		*Il1b*	3.75
		*Il1rn*	3.52
		*Imp4*	1.51
		*Inhbb*	4.81
		*Inpp5e*	1.45
		*Jun*	4
		*Junb*	4.72
		*Kif5b*	1.87
		*Klf6*	2.48
		*L42293*	1.71
		*Leprel1*	1.71
		*Litaf*	2.57
		*Lman2l_predicted*	2.12
		*Lmbr1l*	2.04
		*Lmna*	1.7
		*Lmo2*	1.83
		*Lrg1*	2.66
		*Lrrc59*	1.62
		*Lrrfip1*	1.79
		*Lypd3*	1.7
		*Map3k6_predicted*	3.36
		*Map3k8*	2.57
		*Mapkapk2*	1.73
		*Mcl1*	1.76
		*Mgp*	1.71
		*Mlstd2*	1.63
		*Mt1a*	3.52
		*Myd116*	2.4
		*Nebl_predicted*	1.77
		*Nfkb2*	1.92
		*Nfkbia*	1.9
		*Ng3*	1.68
		*Npff*	1.64
		*Nr4a1*	4.16
		*Nr4a3*	6.53
		*Nupr1*	4.73
		*P4ha1*	3.83
		*Pctk2*	1.6
		*Pdia4*	4.69
		*Pdia6*	1.57
		*Pdlim1*	1.65
		*Phlda1*	3.17
		*Piga*	3.77
		*Pla2g1b*	3.62
		*Plagl1*	3.7
		*Plod1*	1.88
		*Polg2_predicted*	1.92
		*Pom121*	1.77
		*Ppid*	1.87
		*Ppm1d_predicted*	2.23
		*Pprc1_predicted*	1.73
		*Prpf4b*	1.94
		*Pspla1*	2.3
		*Psrc2*	1.76
		*Ptpn1*	1.74
		*Rad18_predicted*	1.66
		*Rangap1*	1.68
		*Rbm15b_predicted*	2.33
		*Rela*	1.48
		*Rgs1*	13.07
		*Rgs2*	2.39
		*Ripk3*	2.38
		*Rnasen*	1.6
		*Rnd1*	6.54
		*Runx1*	2.53
		*Sat*	2.08
		*Sectm1b*	10
		*Serpine1*	10.3
		*Serpinh1*	3.29
		*Slc20a1*	1.57
		*Slc2a5*	2.42
		*Slc38a2*	1.79
		*Slc5a3*	2.17
		*Slc7a1*	2.75
		*Slfn2_predicted*	1.89
		*Slpi*	6.49
		*Snf1lk*	3.51
		*Solt_predicted*	3.47
		*Sphk1*	4.44
		*Spp1*	2.43
		*Spry1_predicted*	2.19
		*Ssfa2_predicted*	1.57
		*St13*	1.81
		*Stat3*	1.68
		*Stip1*	2.66
		*Stk17b*	1.78
		*Syde1_predicted*	1.41
		*Synj2bp*	1.4
		*Tgm2*	2.69
		*Timp1*	3.37
		*Tmem33*	1.87
		*Tnfrsf12a*	4.35
		*Tnfrsf1b*	1.99
		*Tra1_predicted*	2.08
		*Traf6_predicted*	1.56
		*Trib3*	3.51
		*Trip10*	1.59
		*Ubqln1*	1.79
		*Ugdh*	1.69
		*Ung*	1.94
		*Upp1*	3.16
		*Vegfa*	1.56
		*Verge*	22.43
		*Vipr2*	2.26
		*Vpreb3_predicted*	6.01
		*Wtip_predicted*	1.58
		*Xab2*	1.58
		*Zdhhc23*	1.93
		*Zfand2a*	2.5
		*Zfp265*	1.64
		*Zfp36l1*	1.69
		*Zfr*	1.65
		*Znf183*	1.71

**Table 4:** Relative mRNA expression of experimental treatments at 3 hr for genes involved in adherin junctions, tight junctions, or angiogenesis

**NCBI gene symbol**	**AMPH compared to control**		**EIH compared to control**		**AMPH compared to EIH**	
	**MAV**	**Choroid plexus**	**MAV**	**Choroid plexus**	**MAV**	**Choroid plexus**
*Ocln*	**0.36**	1.17^a^	0.67	1.21^a^	0.54	0.96^a^
*Cldn1*	0.86	1.04^a^	0.97	1.18^a^	0.88	0.88^a^
*Cldn3*	1.22	1.02	0.99	0.88	1.23	1.16
*Cldn4*	1.50	0.77	1.60	1.00	0.94	0.78
*Cldn5*	**0.41**^a^	0.32	0.59^a^	0.48	0.69^a^	0.66
*Cldn11*	0.88	0.48	0.81	0.77	1.09	0.62
*Magi2*	0.90	0.51	1.16	0.88	0.77	0.58
*Magi3*	0.74	1.01	1.08	1.07	0.69	0.94
*Esam*	**2.07**^b^	1.92	1.14	1.85	1.85^a^	1.04
*F11r* (Jam1)	0.73^a^	1.09	0.69^a^	0.94	1.06^a^	1.16
*Jam2*	**0.67**	0.97	0.96	0.92	0.70	1.05
*Jam3*	0.77	0.77	0.84	0.71	0.91	1.09
*Cdh5*	0.92	0.87	1.03	1.41	0.90	0.62
*Aqp4*	0.76^a^	0.52	1.05^b^	0.66	0.73^a^	0.78
*Slc2a1*	1.39	0.82	1.13	1.04	1.24	0.79
*Angpt2*	1.02^a^	1.12	1.02^a^	1.21	1.00^a^	0.66
*Tek*	0.43	0.88	0.62	1.38	0.69	0.64
*Ctnnb1*	1.29	0.74	0.83	0.68	1.56	1.09
*Pdgfrb*	0.60	0.60	0.86	0.95	0.70	0.63
*Sox18*	**0.33**^a^	0.70	0.67^a^	0.70	0.50^a^	1.01
*Agrn*	**0.70**	0.81	0.77	0.70	0.91	1.15

Bold text indicates genes that were significantly differentially expressed (FDR = 0.10) between treatment comparisons.

^a ^Array expression results were confirmed with RT-PCR.

^b ^Array expression results were not confirmed with RT-PCR.

**Table 5:** RT-PCR determination of gene expression in MAV, striatum, and parietal cortex

		**3 hr**			**1 day**	
**NCBI gene symbol**	**Brain Region**	**Control**	**AMPH**	**EIH**	**AMPH**	**EIH**
*Angpt2*	MAV	1.08 ± 0.37	0.82 ± 0.23 (0.76)	0.81 ± 0.17 (0.75)	N.D.	N.D.
	Parietal Cortex	0.02 ± 0.004	0.04 ± 0.01 (2.00)	0.03 ± 0.01 (1.50)	N.D.	N.D.
	Striatum	0.03 ± 0.001	0.06 ± 0.02 (2.00)	0.04 ± 0.01 (1.33)	N.D.	N.D.
*Aqp1*	MAV	6.67 ± 1.61	5.30 ± 1.89 (0.79)	5.68 ± 1.11 (0.85)	N.D.	N.D.
	Parietal Cortex	0.01 ± 0.003	0.08 ± 0.17 (8.00)	0.02 ± 0.02 (2.00)	N.D.	N.D.
	Striatum	0.02 ± 0.003	0.04 ± 0.01 (2.00)	0.02 ± 0.003 (1.00)	N.D.	N.D.
*Aqp4*	MAV^b,c^	1.00 ± 0.34	1.11 ± 0.27 (1.11)	1.74 ± 0.11 (1.74)	N.D.	N.D.
	Parietal Cortex	0.75 ± 0.03	0.73 ± 0.12 (0.97)	0.79 ± 0.10 (1.05)	N.D.	N.D.
	Striatum	1.86 ± 0.01	2.14 ± 0.24 (1.15)	2.01 ± 0.36 (1.08)	N.D.	N.D.
*Cldn5*	MAV^a^	3.95 ± 1.18	0.99 ± 0.52 (0.25)	1.53 ± 0.88 (0.39)	N.D.	N.D.
	Parietal Cortex	0.41 ± 0.06	0.19 ± 0.05 (0.46)	0.19 ± 0.12 (0.46)	N.D.	N.D.
	Striatum	0.45 ± 0.04	0.20 ± 0.06 (0.44)	0.28 ± 0.13 (0.62)	N.D.	N.D.
*Esam*	MAV	3.55 ± 1.16	4.29 ± 1.19 (1.21)	2.68 ± 0.42 (0.75)	N.D.	N.D.
	Parietal Cortex	0.25 ± 0.03	0.66 ± 0.14 (2.64)	0.68 ± 0.42 (2.72)	N.D.	N.D.
	Striatum	0.35 ± 0.02	0.80 ± 0.21 (2.29)	0.55 ± 0.04 (1.57)	N.D.	N.D.
*F11r*	MAV^a,b^	1.63 ± 0.27	0.96 ± 0.15 (0.59)	0.96 ± 0.20 (0.59)	N.D.	N.D.
	Parietal Cortex	0.11 ± 0.02	0.10 ± 0.01 (0.91)	0.10 ± 0.03 (0.91)	N.D.	N.D.
	Striatum	0.19 ± 0.02	0.15 ± 0.01 (0.79)	0.16 ± 0.05 (0.84)	N.D.	N.D.
*Fst*	MAV^a,d^	0.11 ± 0.05	2.23 ± 1.33 (20.27)	0.33 ± 0.15 (3.00)	0.25 ± 0.16 (2.78)	0.09 ± 0.02
	Parietal Cortex	0.11 ± 0.05	0.21 ± 0.06 (1.91)	0.22 ± 0.04 (2.00)	N.D.	N.D.
	Striatum	0.19 ± 0.05	0.40 ± 0.20 (2.10)	0.33 ± 0.04 (1.74)	N.D.	N.D.
*Has1*	MAV^a,d^	0.05 ± 0.02	0.20 ± 0.08 (2.0)	0.760 ± 0.670 (15.20)	0.21 ± 0.07 (1.75)	0.12 ± 0.02
	Parietal Cortex	0.04 ± 0.01	0.11 ± 0.03 (2.75)	0.13 ± 0.04 (3.25)	N.D.	N.D.
	Striatum	0.03 ± 0.01	0.07 ± 0.03 (2.33)	0.06 ± 0.01 (2.00)	N.D.	N.D.
*Lbp*	MAV^d^	2.22 ± 0.86	3.52 ± 2.43 (1.58)	1.40 ± 0.48 (0.63)	12.05 ± 7.42 (5.06)	2.38 ± 1.12
*Reg3a*	MAV^a,d^	0.001 ± 0.001	0.45 ± 0.50 (450.00)	0.01 ± 0.01 (10.00)	N.D.	N.D.
	Parietal Cortex	0.001 ± 0.001	0.002 ± 0.001 (2.00)	0.005 ± 0.00 (5.00)	N.D.	N.D.
	Striatum	0.01 ± 0.01	0.02 ± 0.01 (2.00)	0.03 ± 0.004 (3.00)	N.D.	N.D.
*Reg3b*	MAV^a,d^	0.001 ± 0.001	0.21 ± 0.27 (210.00)	0.01 ± 0.004 (10.00)	0.44 ± 0.36 (62.86)	0.007 ± 0.014
	Parietal Cortex	0.001 ± 0.00	0.001 ± 0.00 (1.00)	0.001 ± 0.00 (1.00)	N.D.	N.D.
	Striatum	0.0005 ± 0.0001	0.0007 ± 0.0002 (1.40)	0.0005 ± 0.0003 (1.00)	N.D.	N.D.
*Reg3g*	MAV^a^	0.003 ± 0.002	0.31 ± 0.33 (103.33)	0.02 ± 0.01 (6.67)	0.19 ± 0.26 (47.50)	0.004 ± 0.005
*Sct*	MAV^a,d^	0.004 ± 0.001	0.025 ± 0.017 (6.25)	0.006 ± 0.002 (1.50)	0.014 ± 0.004 (3.50)	0.004 ± 0.002
*Slc15a1*	MAV^a,c^	0.19 ± 0.09	3.19 ± 5.72 (16.79)	0.16 ± 0.07 (0.84)	0.04 ± 0.04 (1.33)	**0.03 ± 0.02**
	Parietal Cortex	0.03 ± 0.003	0.04 ± 0.01 (1.33)	0.03 ± 0.003 (1.00)	N.D.	N.D.
	Striatum	0.04 ± 0.01	0.05 ± 0.01 (1.25)	0.04 ± 0.01 (1.00)	N.D.	N.D.
*Sox18*	MAV^a^	5.57 ± 2.58	0.96 ± 0.33 (0.17)	2.11 ± 1.54 (0.38)	N.D.	N.D.
	Parietal Cortex	0.36 ± 0.06	0.17 ± 0.07 (0.47)	0.24 ± 0.20 (0.67)	N.D.	N.D.
	Striatum	0.46 ± 0.10	0.23 ± 0.08 (0.50)	0.26 ± 0.25 (0.56)	N.D.	N.D.

All genes are expressed as a percentage of *Gapdh *and are shown as the mean plus/minus standard error of the mean. Fold-changes of treatment to control are given in parentheses at the 3 hr time point. At the 1 day time point, fold-changes (in parentheses) are expressed as AMPH to EIH. All the RT-PCR primers appeared to function optimally except those for *Slc15a1 *which gave a somewhat bimodal melt curve (several sets of primers were tried). The number of RNA aliquots (one per animal) used to determine expression per treatment group were as follows: 3 hr control, n = 6; 3 hr AMPH, n = 7; 3 hr EIH, n = 6; 1 day AMPH, n = 6; 1 day EIH, n = 6.

N.D. designates expression not determined.

Symbols indicating statistically significant difference between treatments are found as a superscript after the brain region (second column).

^a ^AMPH significantly differs from control at 3 hr (p < 0.05).

^b ^EIH significantly differs from control at 3 hr (p < 0.05).

^c ^AMPH significantly differs from EIH at 3 hr (p < 0.05).

^d ^AMPH significantly differs from EIH at 1 day (p < 0.05).

The expression of *Ctgf* and *Timp1,* which are genes related to angiogenesis, did increase in the choroid plexus after AMPH like it did in the MAV (Table 3, [24] and Additional file 3: Table S3). The decreases in the angiogenesis-related or tissue remodeling genes *Mmp2*, *Mmp14*, and *Mmp16* were not significant (Table 3) and not as prominent in the choroid plexus as the MAV (Additional file 3: Table S3, Additional file 4: Table S4 and [24]). The ERSR response from AMPH in the choroid plexus was similar to MAV, with multi-fold increases seen in *Hspa5*, *Atf3, Atf4, Nfkbia,* and *Nfkb2* (Table 3, Additional file 3: Table S3). As well, expression of the protein di-isomerases encoding genes *Pdia3, Pdia4,* and *Pdia6* was increased similarly by AMPH and EIH in both MAV and choroid plexus (Table 3, Additional file 3: Table S3 and Additional file 4: Table S4).

#### Effects of AMPH and EIH on genes in MAV that are not directly related to vasculature

Array expression data indicated significant expression changes within the MAV due to either EIH or AMPH when compared to control at the 3 hr time point (Additional file 3: Table S3). At the 3 hr time point, significant differences were found in the expression level of 265 genes (FDR = 0.10) when comparing EIH/control and 1,129 genes (FDR = 0.10) when comparing AMPH/control. Core analysis with the IPA software found that the gene expression patterns clearly indicate that both EIH and AMPH produced significant increases in genes involved in reactive oxygen species (ROS) and inflammatory responses with some indication that apoptosis may have occurred (data not shown). When comparing AMPH to EIH at 3 hr, a total of 70 genes (FDR = 0.10) were differentially expressed (Table 6). IPA analysis suggests that these genes are involved in cancer, reproductive system disease, gastrointestinal, cardiovascular, and immunological inflammatory diseases.

**Table 6:** Genes with significant differential expression between AMPH and EIH at 3 hr in MAV

**NCBI gene symbol**^**a**^	**AMPH/EIH**	**Specific gene function**	**General function**	**Reference**
*A3galt2*	2		T-cell function?	*NCBI Entrez Gene*
*Abca1*	2.18	ATP-binding cassette (ABC) transporters	Cholesterol transport	*NCBI Entrez Gene*
*Actn3*	2.47	Cross links actin containing filaments		*NCBI Entrez Gene*
*Ada*	0.41	Adenosine deaminase	Immune system – modulates lymphocyte activity?	*NCBI Entrez Gene*
*Adamts9*	2.97	Metalloproteinase inhibitor	Vascular Function - angiogenesis?	PMID: 19052845
*Akr1b8*	4.49	Unknown	Carcinogen-dependent tumor-associated protein	*NCBI Entrez Gene*
*Asns*	2.22	Asparagine synthesis	Cell growth and differentiation – blocks cell proliferation	*NCBI Entrez Gene*
*Bzw2*	2.04	Transcription factor?		*NCBI Entrez Gene*
*Cd14*	3.14	Binds bacterial lipopolysaccharides	Cell surface protein that mediates monocyte responses to bacterial lipopolysaccharides	*NCBI Entrez Gene*
*Cd93*	1.75		Immune system function?	*NCBI Entrez Gene*
*Cyp1b1*	2.61	Cytochrome P450 monooxygenases	Lipid metabolism -polycyclic aromatic hydrocarbon, steroid metabolism or phospholipids metabolism	*NCBI Entrez Gene*
*Egfr*	2.01	Epidermal growth-factor receptor	Cell growth and differentiation	*NCBI Entrez Gene*
*Eif2b3*	1.75	Subunit of initiation factor eIF2B		*NCBI Entrez Gene*
*Emp1*	1.88	Epithelial membrane protein 1		*NCBI Entrez Gene*
*Enpp3*	3.10	Hydrolysis of extracellular nucleotides	Unknown - Found in immature glia, gut and pancreas	*NCBI Entrez Gene*
*Fcgr3*	1.86	Receptor on Fc portion of IgG	Immune system function	*NCBI Entrez Gene*
*Fgl2*	3.11	Regulates thrombosis	Vascular endothelium regulation of thrombosis	PMID: 15100314
*Fmo5*	4.03	N-oxidation of amino-trimethylamine	Fmo5 not well characterized	*NCBI Entrez Gene*
*Foxj1*	0.41	Unclear in mammals	Unknown – characterization incomplete	*NCBI Entrez Gene*
*Fst*^b^	6.64	Can Inhibit vesicular release of FSH	Adipogenesis? Cancer?	*NCBI Entrez Gene *PMID19470636
*Gda*	2.53	Hydrolytic deamination of guanine	Cell signaling? – incomplete characterization	*NCBI Entrez Gene*
*Gja5*	2.57	Endothelial gap junction protein	Vascular function - structural integrity	*NCBI Entrez Gene*
*Gpr4*	2.20	GCP receptor for lysophosphatidylcholine	May mediate monocyte transmigration through endothelial	*NCBI Entrez Gene, *PMID17364894
*Has1*^b^	7.20	Regulates the biosynthesis of hyaluronan	Immune System - inflammation related?	PMID: 17611197
Ilr2*	5.48	Interleukin 1 receptor family	Inhibits the activity of Il-1	*NCBI Entrez Gene*
Ilrn*	3.35	Binds to Interleukin 1 receptor	Inhibits the effects of IL-1	*NCBI Entrez Gene*
*Itpkc*	2.21	Phosphorylates inositol triphosphate		*NCBI Entrez Gene*
*Lbp*	4.15	Binds LPS and interacts with the CD14 receptor	Acute-phase immunologic response to gram-negative bacterial infections	*NCBI Entrez Gene*
*Lrrc4c*	0.38	Lrrc4c (NGL1) is a specific binding partner for netrin G1	Has been implicated in axonal guidance	*NCBI Entrez Gene*
*Llgl1*	2.14		Non-muscle myosin II heavy chain and a kinase associated	*NCBI Entrez Gene*
*Mt1a*^b^	2.77	Protects against oxidative stress	Cardiovascular function - protection from ROS in diabetes?	PMID: 18249147 PMID: 18349110
*Mthfd1l*	1.94	Synthesis of tetrahydrofolate in the mitochondrion		*NCBI Entrez Gene*
*Ndrg1*	2.21	Cytoplasmic protein involved in stress	Involved in endothelial cell migration	PMID: 19760510
*Nef3*	0.45	Medium neurofilament protein	Biomarker of neuronal damage	*NCBI Entrez Gene*
*Nnij1*	1.67		Nerve injury associated protein	*NCBI Entrez Gene*
*Ogfrl1*	1.93	Opioid growth factor receptor-like 1		*NCBI Entrez Gene*
*Olfm3*	0.42		Associated with lung cancer	*NCBI Entrez Gene*
*Pmp22*	1.79		Component of myelin in the peripheral nervous system	*NCBI Entrez Gene*
*Pstpip1*	1.76		Immune system-related	*NCBI Entrez Gene*
*Ptprj*	1.89	Protein tyrosine phosphatase	Cell cycle and differentiation	*NCBI Entrez Gene*
*Ptges*	3.02	Synthesized PGE2	Vascular Function - vasodilatation	*NCBI Entrez Gene*
*Ramp3*	2.97	Calcitonin receptor modulating protein	Angiogenesis and vascular integrity	PMID: 18097473
*Reg3a*	23.49	Extracellular matrix repair?	Inflammation – expressed in pancreatitis and liver cancer damage - function unknown in MAV	*NCBI Entrez Gene*
*Reg3b*	27.01	Extracellular matrix repair?	Inflammation – related to pancreatic damage - function unknown in MAV	PMID: 19077460
*Reg3g*	8.29	Extracellular matrix repair?	Inflammation – related to pancreatic damage and development - function unknown in MAV	*NCBI Entrez Gene*
*Rrp9*	1.77		Processing of 18 s rRNA during ribosome synthesis	*NCBI Entrez Gene*
*Rsad2*	2.27		Bone formation?	*NCBI Entrez Gene*
*Sct*	4.93	Induces gastric relaxation	Endocrine hormone in pancreas and gut – function in MAV may be stimulate bicarbonate secretion	*NCBI Entrez Gene*
*Slc15a1*	33.90	Hydrogen peptide co-transporter	Inflammation? – transports polypeptides in intestine - function unknown in MAV	*NCBI Entrez Gene *PMID: 19462432
Slco4a1*	1.85	Organic anion transporter		*NCBI Entrez Gene*
Slpi*	6.58		Protects epithelial tissues from serine proteases	*NCBI Entrez Gene*
*Timp1*	2.60	Metallopeptidase inhibitor	Multiple functions some vascular-related	*NCBI Entrez Gene*
*Tmem2*	2.72	Membrane spanning protein?	Unknown- characterization incomplete	*NCBI Entrez Gene*
*Tmem63c*	2.02	Membrane spanning protein?	Unknown- characterization incomplete	*NCBI Entrez Gene*
*Wnt4*	1.73		Cell signaling and development	*NCBI Entrez Gene*

PMID: denotes the Pub Med ID if a particular publication has been primarily used to determine specific and general gene functions.

^a ^All listed genes were differentially affected by AMPH compared to EIH (FDR = 0.10). Also, genes with very high expression in blood (data not shown) are excluded from this table as well as genes which were previously reported to be affected [24,25].

^b ^Relative to control, both EIH and AMPH significantly increased expression.

* Indicates some expression may be due to residual blood in MAV.

Three genes, linked to pancreatitis and cancer, the regenerating islet-derived 3 alpha, beta and gamma (*Reg3a*, *Reg3b* and *Reg3g*), were increased by more than 100-fold by AMPH within 3 hr, but not by EIH (Table 5 and Table 6). These genes were not significantly affected in the choroid plexus at the 3 hr time point (Table 5 and Table 7). At 1 day after AMPH, *Reg3a, Reg3b,* and *Reg3g* expression was still elevated in the MAV (Table 5). Our data also indicated that the intestinal hydrogen peptide co-transporter (*Slc15a1),* follistatin *(Fst),* hyaluronan synthase (*Has1)*, secretin *(Sct)* and lipopolysaccharide binding protein *(Lbp)* genes were also increased to a much greater extent by AMPH than EIH in the MAV (Table 6). The significance of the increases in these genes at the 3 hr time point was verified with RT-PCR, with the exception of *Lbp* (Table 5). At the 1 day time point, differences between EIH and AMPH in the expression of *Slc15a1, Fst,* and *Sct* were lesser in fold and not deemed statistically significant. However, RT-PCR data indicated that the expression levels of *Sct*, *Has1*, *Fst,* and particularly *Lbp* were significantly greater after AMPH compared to EIH at the 1 day time point (Table 5).

**Table 7:** RT-PCR determination of gene expression in the choroid plexus

	**3 hr**			**1 day**	
**NCBI gene symbol**	**Control**	**AMPH**	**EIH**	**AMPH**	**EIH**
*Apold1*(Verge)^*a*^	0.03 ± 0.002	0.06 ± 0.02 (2.00)	0.06 ± 0.07 (2.00)	N.D.	N.D.
*Bmp4*^*a,c*^	0.55 ± 0.04	3.65 ± 1.26 (6.64)	0.84 ± 0.23 (1.53)	N.D.	N.D.
*Cebpd*^a,b^	0.01 ± 0.003	0.24 ± 0.09 (24.00)	0.15 ± 0.13 (15.00)	N.D.	N.D.
*Cldn1*	5.74 ± 0.70	5.73 ± 0.98 (1.00)	6.49 ± 0.80 (1.13)	N.D.	N.D.
*Cxcl1*^*a,b*^	0.004 ± 0.002	0.40 ± 0.62 (100.00)	0.30 ± 0.51 (75.00)	N.D.	N.D.
*Cyr61*^*a,b*^	0.24 ± 0.06	3.54 ± 2.70 (14.75)	3.16 ± 5.10 (13.17)	N.D.	N.D.
*Dio2*^a^	0.01 ± 0.005	4.34 ± 2.03 (434.00)	1.08 ± 1.13 (108.00)	N.D.	N.D.
*Edn1*^b^	0.003 ± 0.001	0.005 ± 0.002 (1.67)	0.011 ± 0.010 (3.67)	0.003 ± 0.004 (1.50)	0.002 ± 0.000
*Fst*^b,d^	0.03 ± 0.02	0.06 ± 0.02 (2.00)	0.10 ± 0.06 (3.33)	0.03 ± 0.02 (3.00)	0.01 ± 0.003
*Has1*^a,b^	0.09 ± 0.06	0.17 ± 0.03 (1.89)	0.17 ± 0.02 (1.89)	0.11 ± 0.02 (1.10)	0.10 ± 0.02
*Lbp*^a^	0.11 ± 0.06	0.53 ± 0.32 (4.81)	0.24 ± 0.12 (2.18)	0.97 ± 0.57 (2.11)	0.46 ± 0.31
*Nat3*^b^	0.01 ± 0.004	0.01 ± 0.004 (1.00)	0.01 ± 0.008 (1.00)	0.004 ± 0.003 (0.50)	0.008 ± 0.010
*Nos3*	0.06 ± 0.03	0.09 ± 0.04 (1.50)	0.10 ± 0.06 (1.67)	0.08 ± 0.05 (1.33)	0.06 ± 0.01
*Ocln*^a^	4.96 ± 0.52	7.24 ± 1.12 (1.46)	6.01 ± 1.76 (1.21)	N.D.	N.D.
*Reg3b*	N.E.	N.E.	N.E.	N.E.	N.E.
*Sct*	N.E.	N.E.	N.E.	N.E.	N.E.
*Sectm1b*^a^	0.01 ± 0.002	0.46 ± 0.15 (46.00)	0.15 ± 0.14 (15.00)	N.D.	N.D.

All genes are expressed as a percentage of *Gapdh, *and are shown as the mean plus/minus standard error of the mean. Fold-changes of treatment to control are given in parentheses at the 3 hr time point. At the 1 day time point, fold-changes (in parentheses) are expressed as AMPH to EIH. The number of RNA aliquots (two to three animals per aliquot) used to determine expression per treatment group were as follows: 3 hr control, n = 7; 3 hr AMPH, n = 7; 3 hr EIH, n = 7; 1 day AMPH, n = 8; 1 day EIH, n = 7.

N.E. means gene not expressed (mean Ct value > 35).

N.D. designates expression not determined.

Symbols indicating statistically significant difference between treatments are found as a superscript after the gene name (first column).

^a ^AMPH significantly differs from control at 3 hr (p < 0.05).

^b ^EIH significantly differs from control at 3 hr (p < 0.05).

^c ^AMPH significantly differs from EIH at 3 hr (p < 0.05).

^d ^AMPH significantly differs from EIH at 1 day (p < 0.05).

Approximately 272 genes, or 2.5% of the total number of all the genes present on the oligo array, had 15-fold or higher expression levels in the MAV relative to both the striatum and parietal cortex (Additional file 2: Table S2). Using this proportion, the expected number of differentially expressed genes with high expression in the MAV at the 3 hr time (when comparing EIH/control and AMPH/control) was computed. The expected (actual) counts for EIH/control and AMPH/control are 7 (13) and 28 (61), respectively. When comparing AMPH to EIH, two of the differentially expressed genes were expected to have high expression in MAV; however, the actual count is 7. Therefore, we found the observed counts to be higher than the expected counts in all three pairwise treatment comparisons.

### Effects of AMPH and EIH on genes not directly related to vasculature in choroid plexus

Our choroid plexus oligo array data show that, at the 3 hr time point, EIH significantly affected the expression of 208 genes (FDR = 0.10) when comparing EIH to control, while 73 genes differed between AMPH and control (FDR = 0.10, Table 3). The gene expression patterns indicate that both EIH and AMPH produced significant similar increases in genes involved in ROS, inflammatory responses and damage. Although relative to control both AMPH and EIH dramatically altered gene expression in the choroid plexus at the 3 hr time point, when the two treatments were compared to each other, no genes (FDR = 0.10) were found to be differentially affected (Table 3). This would indicate that both AMPH and EIH had very similar effects on choroid function at 3 hr. However, the sample size of four used in the array analysis may have contributed for the lack of significant genes which were differentially affected by AMPH compared to EIH. Several genes with the greatest differential expression in AMPH compared to EIH, including *Apold1* (Verg), *Bmp4, Cebpd, Cxcl1,Cyr61, Dio2, Fst, Has1, Lbp,* and *Sectm1b*, were further evaluated using RT-PCR. RT-PCR analysis, using a larger sample size (n = 7 to 8), determined that there was a 2-fold or more increase with AMPH compared to EIH in *Bmp4*, *Dio2, Lbp,* and *Sectm1b* at 3 hr (Table 7). One day *Lbp* levels were 4- and 8 -fold greater than the control *Lbp* at the 3 hr time point. Array analysis detected only three genes (*Nat3*, *Angel2* and *Arsk*; FDR = 0.20) to be significantly differentially affected by AMPH compared to EIH at 1 day. The *Nat3* increase was potentially the most significantly affected by AMPH relative to EIH at 1 day by array analysis but RT-PCR analysis failed to verify this finding. RT-PCR was not used to verify *Angel2* and *Arsk* expression since there was less than a 1.5-fold (AMPH/EIH) change for both genes. Additionally, RT-PCR analysis indicated that *Lbp* expression in AMPH was 2-fold higher at the 1 day time point compared to EIH but the difference was not found to be significant.

## Discussion

New data have been presented that compares the global gene expression (at the mRNA level) in the choroid plexus and the MAV under control conditions and after exposure to EIH or a neurotoxic AMPH exposure. Under control conditions, the high degree of similarity in the global gene expression profiles of the MAV and choroid plexus suggests some overlap in their various physiological roles supporting brain function [2-6,11,13], particularly the CSF. However, basal expression of genes related to the BBB and other tight junctions show some distinct differences in MAV versus choroid plexus. Expression changes related to genes involved in oxidative stress, ERSR, and the immune system indicated that both the choroid plexus and MAV are adversely affected by AMPH and EIH. However, the expression response in the MAV to neurotoxic doses of AMPH and EIH (to a lesser extent) was more pronounced than in the choroid plexus with respect to genes controlling vascular tone and angiogenesis. Unique genes were found in the choroid plexus and in the MAV that give further insight into differences between AMPH and EIH effects and may serve as biomarkers of damage.

### Comparisons of choroid plexus and MAV expression in control

In our study, there was a high correlation of r = 0.92 between gene expression in the choroid plexus and MAV for control animals. Furthermore, 50% of the genes that had a 15-fold (60% for >10-fold) or greater enrichment (compared to striatum and parietal cortex) in choroid plexus also had a 15-fold or higher enrichment in the MAV, indicating a likely possibility of some overlap in functional capabilities. Over 70% of the overlapping highly expressed genes between choroid plexus and MAV could be categorized into groups involved in: 1) extracellular matrix and cell-cell junctions, 2) development and transcription regulation, 3) ion and solute transport and homeostasis, 4) vasculature and heart, and 5) immune system (Table 1 and Additional file 2: Table S2). The majority of the rest of the genes were lumped into an unknown and miscellaneous category. Since 50% of these genes have an unknown function, the percentage of genes actually in the top 5 categories could be as high as 85%. The MAV contained many more genes in the retinoic acid & lipid processing category than did the choroid plexus.

Many of the highly expressed genes identified in the MAV that are placed in the ion and solute transport category may play a role in the pial and arachnoid membrane function of the MAV. Many of these genes are also expressed highly in the choroid plexus and therefore could play a role in ion and solute composition regulation of CSF (Table 1 and Additional file 2: Table S2). Genes highly expressed in the MAV and choroid plexus that are related to water, ion, and solute homeostasis include the water channel *Aqp1*[41], the sodium bicarbonate co-transporter *Slc4a5*[42], the chloride/bicarbonate exchanger *Slc26a7*[43], the potassium inwardly-rectifying channel *Kcnj13*[44], the organic anion transporter *Slco1a5*[45], and two transporters that appear to be related to the regulation of thyroid function, solute carrier family 5, sodium iodide symporter, member 5 (*Slc5a5*[46] and transthyretin (*Ttr*)) [47]. Most of these genes have also been previously identified in the epithelial cells of the kidney, pancreas, lung, and placenta.

Many genes with relatively high expression in the MAV (*e.g.*, annexin A1, *Anxa1*[48]; annexin A2, *Anxa2*[46]; major histocompatibility complex class II invariant chain Cd74 molecule, *Cd74*[49]; lectin galactoside-binding soluble 1, *Lgals1*, [50]; defensin beta 1, *Defb1*[51]; chemokine (C-X-C motif) ligand 14, *Cxcl14*[52]) were identified as important for endothelial cell interactions with myeloid or lymphoid interactions, and could be involved in regulating myeloid and lymphoid cell responses and trafficking in the MAV vasculature and/or the CSF. This suggests that under control conditions the immune surveillance and other functions in the MAV are possibly more extensive than in the cortex and striatum and probably most of the remaining forebrain. Of the remaining genes with relatively high expression in the MAV and choroid plexus, most were related to developmental and transcriptional regulation. However, except for possibly those genes involved in the insulin-like growth factor pathways, it is not clear what the exact transcription regulatory role of these genes is in the MAV and choroid plexus. Also, it is not clear whether the translation products of all these genes play a role in just MAV and choroid plexus development and function or whether some are “released” into the CSF to affect other regions of the brain.

There were twice as many genes (17 versus 8) grouped in the retinoic acid and lipid processing category in the MAV versus the choroid plexus, but the significance of this finding is not clear. There is an even more disparate distribution of genes related to the BBB and tight junctions in the choroid plexus versus the MAV. Even when the 5- to 10-fold greater levels of vasculature present in MAV are taken into account, its levels of BBB-related genes (determined from [53]) were somewhat similar to that of striatum and parietal cortex. The exceptions are *Cldn3, Tjp2* (ZO-2), and *Aqp4*, which may have lower expression levels in the MAV than expected. Thus, it appears that a BBB-related structure is also present in the vasculature of the MAV. The presence of a BBB-like barrier in the MAV would not only protect the surface of the cortex from small molecules and ions present in the blood, but it would also prevent them from leaking into the cerebral spinal fluid and altering its composition. Tight-junction and some BBB-related gene expressions are present in the choroid plexus but differ from the brain proper [6,8]. Our expression data indicates that *Ocln, Cldn1, Cldn3, Cldn9, Crb3, Tjp-3, F11r* (Jam1), and *Jam3* would be some of the more important genes involved in choroid plexus tight-junctions.

### Effects of AMPH and EIH on gene expression in choroid plexus compared to MAV

Gene expression responses to the adverse effects of EIH and AMPH differed to some extent between the MAV and choroid plexus. In the choroid plexus, genes regulating vascular tone, such as *Nos3, Edn1,* and *Vip*, were not significantly affected by AMPH or EIH but they were greatly altered in the MAV [24]. However, *Adora1a* and *Adora1b* were elevated in choroid plexus, but not the MAV, and may affect vasodilatation in response to AMPH or EIH in this region. Also, some of the genes involved in angiogenesis (*e.g.*, *Ctgf* and *Timp*) were significantly increased by AMPH and EIH in the choroid plexus, just as they were in MAV. However, other genes involved in angiogenesis that were decreased in the MAV by AMPH (*Akt1, Anpep, Mmp2, Mmp14,* and *Mmp16*) were not significantly affected in the choroid plexus. On the other hand, genes related to protein processing such as heat-shock proteins, protein disulfide isomerases, and the ERSR responded very similarly to AMPH and EIH in the choroid plexus and MAV.

We have placed particular emphasis on evaluating expression changes related to the BBB since loss of BBB integrity can have severe damaging effects on the brain [20-23]. With respect to genes related to BBB and tight junctions, there were differences in response to AMPH and EIH between the choroid plexus and MAV. Occludin and the claudins are integral membrane proteins, components of tight junction strands, and are necessary for proper BBB development and integrity [54,55]. Our data show that in the MAV, both *Ocln* and *Cldn5* are well expressed and down regulated significantly by EIH and even more so by AMPH. However, in the choroid plexus, *Ocln* expression was slightly increased by AMPH. Although *Cldn5* expression was down regulated in the choroid plexus, its constitutive expression levels, as well as *Cldn11*, were very low. Also, *F11r* (Jam1) and *Jam2,* which are key components regulating BBB [56-58], were significantly decreased by both EIH and AMPH in the MAV but were not significantly affected in choroid plexus. Overall, AMPH and EIH had a more disruptive effect on BBB/tight junction gene expression in the MAV than in the choroid plexus. It is not clear whether this is the result of the temporary suspension of angiogenesis during the insult by AMPH and EIH, which may be a protective response if short in duration.

The genes in the MAV with the largest expression increases resulting from AMPH exposure were somewhat unexpected since they do not appear to be directly related to vasculature but rather to damage, inflammation, or infection in other organs such as pancreas. These genes include *Reg3a, Reg3b, Reg3g, Slc15a1, Sct, Fst,* and *Lbp*, and are potential biomarkers for the damaging effects of AMPH in MAV since they are affected to a much lesser extent by EIH. *Reg3a, Reg3b*, *Reg3g,* and *Lbp* expression increases are as, or even more, pronounced at 1 day than at 3 hr after AMPH. The three regenerating genes *Reg3a, Reg3b,* and *Reg3g* are particularly intriguing since they are associated with repair and regeneration of pancreatic tissue [59]. *Reg3a, Reg3b* and *Reg3g* are also involved in anti-bacterial function in intestinal epithelial cells [60] and could be involved in repairing AMPH-induced damage in the MAV. The tremendous increase observed in the expression in the regenerating genes, *Slc15a1, Sct,* and *Fst* after AMPH in the MAV was not observed in the choroid plexus. Increases are seen in *Lbp* expression after AMPH (but very little with EIH) in both the MAV and choroid plexus at 3 hr and 1 day. These changes are also particularly intriguing since *Lbp* expression and protein function is relevant to infection and damage. Together with the bactericidal permeability-increasing protein (BPI), the encoded protein binds LPS and interacts with the CD14 receptor, which probably plays a role in regulating LPS-dependent monocyte responses [61]. Exactly why the increase in *Lbp* gene expression occurs primarily with AMPH but not EIH is unknown. Increases in the AMPH-induced *Lbp* gene expression are more pronounced in MAV compared to choroid plexus and are reflective of AMPH producing a much greater effect on genes related to infection and inflammation than does EIH.

Bone morphogenic protein 4 (*Bmp4*) and to a lesser extent secreted and transmembrane protein 1A and 1B (*Sectm1b*) were the only genes that had a greater change (increase) in expression in the choroid plexus after AMPH but not after EIH. Not much is known about Bmp4 effects on the choroid plexus so it is unclear whether this effect would be beneficial. On the other hand, if the protein product were to be secreted into the lateral ventricles, it would likely suppress the progenitor cells present and possibly be detrimental to differentiated neurons and glia surrounding the ventricles [60,62,63]. Little is known as to what *Sectm1b* increases would translate to in the choroid plexus because very little is known about this gene in rodents and its homolog in humans. Nonetheless, its 40-fold mRNA increase in choroid plexus after the AMPH treatment would likely have some effect if it leads to significant protein increases.

## Conclusions

The comparison of gene expression data in the striatum and parietal cortex, which are two neuronal-rich regions of the brain, to that of the MAV and the choroid plexus under control conditions resulted in a list of more than 100 genes with a much higher expression (15-fold or more) in both the choroid plexus and MAV than in the two neuronal-rich regions. There is a 50% overlap between the highly expressed genes in the choroid plexus with those in the MAV, suggesting an analogy in some of the various physiological functions that the choroid plexus and MAV play in supporting brain function [2-6,26,28], in particular those related to the regulation of ion, solute, and immune processes involving CSF. The global gene expression profile in the MAV indicates that genes related to or regulating tight junctions are important to its vasculature function and to help maintain BBB integrity within the brain. Although EIH affects genes related to the ERSR pathways and ROS in the MAV, it has a less pronounced effect than AMPH on genes involved in ion and solute transport, lipid metabolism, cell survival, and damage/inflammation. Changes in the expression in genes related to vascular tone, angiogenesis, BBB-related and immune-related genes indicate that the adverse vascular effects of AMPH are somewhat more pronounced in the MAV than they are in the choroid plexus. The gene expression changes evoked by AMPH or EIH appear to depict the homeostasis of blood flow and CSF needed to prevent or repair damage in the MAV and choroid plexus.

## Methods

### Animals and experimental design

In brief, 75 to 90-day-old male Sprague–Dawley rats were obtained from the breeding colony of the National Center for Toxicological Research (NCTR). The studies were carried out in accordance with the declaration of Helsinki and the Guide for the Care and Use of Laboratory Animals as adopted and promulgated by the National Institutes of Health. AMPH and EIH exposures were similar to that of Bowyer *et al.*[64] and the overall dosing and experimental design was modeled after previous work [24,25]. Oligo array and RT-PCR data for the choroid plexus was generated in this study; however, a portion of the oligo array data for MAV was presented in Thomas *et al.*[25]. Rats were housed two per cage until three days prior to AMPH exposure, with food and water available *ad libitum*, and were on a daily 12-hr light–dark cycle, with lights on 6:00 a.m. Temperature and humidity were controlled at 23°C and 53%, respectively. During exposure to AMPH, rats were housed one per cage to facilitate behavioral observations without inter-animal interactions. Dosing commenced between 7:30 and 8:00 a.m. and the D-amphetamine sulfate (Sigma-Aldrich, St. Louis, MO, USA) was dissolved in normal saline prior to injection. Control animals received normal saline alone at the same volumes. All doses were administered subcutaneously. The rectal temperature was monitored every hour in all animals for at least 8 hr after the start of dosing. Details of body temperature monitoring and overall experimental design can be found in Thomas et al. [24,25].

### Rats dosed for generating RNA for MAV oligo array analysis

Total RNA for oligo array analysis of MAV gene expression was generated in our previous two studies [24,25]. In these studies, 15 rats were administered four doses of 1 ml/kg normal saline with 2 hr between each dose. Four were given saline at a 24°C room temperature and remained at normal body temperature during body temperature monitoring (normothermic group). Eleven animals were given saline in a 39° to 40°C environment (EIH group, 10 rats survived) and had a hyperthermic profile very similar to the rats given AMPH. Seventeen rats were given four doses of AMPH comprised of a sequential exposure to 5, 7.5, 10 and 10 mg/kg AMPH with 2 hr between each dose (15 rats survived).

### Rats dosed for generating RNA for choroid plexus oligo array and RT-PCR analysis

In order to carry out the oligo array analysis for the choroid plexus data and RT-PCR verification of MAV and choroid plexus mRNA expression levels, an additional 65 animals were dosed specifically for the present study with saline under normothermic conditions (n = 13), saline under EIH conditions (n = 24), or AMPH (n = 28). The trunk blood from these animals was also subjected to oligo array analysis. Blood expression data was used only to determine which genes were present at much higher levels (5-fold or greater) under control conditions in blood compared to MAV and choroid plexus. Such genes were excluded from analysis in MAV and choroid plexus since the expression levels could have been greatly affected by the residual blood present in MAV and choroid plexus.

### Sacrifice and tissue harvest for oligo array data collection

Rats were given an overdose of 300 mg/kg body weight of pentobarbital resulting in deep anesthesia in less than 3 min and were then killed by decapitation. Their brains were then rapidly, but carefully, removed and chilled in ice-cold normal saline for 5 min. The arachnoid and pial membranes of the meninges and associated vasculature surrounding the forebrain were then dissected away from the brain surface (MAV tissues). For all animals used in this study, the parietal cortex, followed by the choroid plexus and striatum were dissected bilaterally on ice and saved for analysis as previously described [24,39].

The array analysis for MAV, striatum, and parietal cortex was performed on total RNA obtained from animals sacrificed in previous work [14,15], with the exception that RNA from one additional animal sacrificed 3 hr after AMPH was added and one less 3 hr EIH sample was available (RNA was depleted by previous RT-PCR studies) [24,25]. Thus, the number of RNA aliquots from individual animals at 3 hr after the final dose was n = 4 for saline at room temperature, n = 4 for EIH, and n = 7 for AMPH. The number of RNA aliquots from individual animals at 1 day after the final dose was n = 5 for EIH and n = 8 for AMPH. For the choroid plexus oligo array and RT-PCR analysis, we used tissue from 15 to 21 rats for each of the five treatment groups in the study (saline 3 h, EIH 3 h, AMPH 3 h, EIH 1d, and AMPH 1d). Unlike the MAV, RNA from choroid plexus was pooled from two to three animals for each aliquot (n = 1) tested. This was done because of the very small amount of choroid plexus tissue that can be harvested from an individual rat (1.5 to 2.0 mg/rat). The pooling resulted in 5 to 7 final separate RNA aliquots to be analyzed by RT-PCR (all aliquots) and oligo arrays (4 aliquots for each treatment). Further details of the experimental groups and animal IDs used for oligo array analysis can be found in GSE23093 (MAV, striatum, and parietal cortex) and GSE29733 (choroid plexus) at the NCBI GEO repository. Note that RNA sample pools, in addition to those listed for MAV and choroid plexus in the NCBI GEO files, were used for the RT-PCR analysis of expression in these brain regions.

### RNA isolation and oligonucleotide array analysis

Tri Reagent (Molecular Research Center Inc., Cincinnati, OH) [65] was used to isolate total RNA with modifications similar to those previously described [24,25]. The final total cellular RNA concentrations (in RNase-free water, 1 mM EDTA) for MAV and choroid plexus ranged from 1.3 to 2 μg/μl, while those of the striatum and parietal cortex ranged from 2.5 to 4.0 μg/μl. The isolated RNA was stored at −70°C until amplification for oligo array analysis. Changes in gene expression were evaluated from total RNA using the Agilent One-Color Microarray-Based Gene Expression Platform. For hybridization onto Agilent-014879 Whole Rat Genome 4 × 44 K 60mer oligonucleotide arrays (G4131F, Agilent Technologies, Palo Alto, CA; NCBI GEO Accession # GPL7294), 500 ng of total RNA was used in the Agilent Quick Amp Labeling Kit (5190–0442) according to the manufacturer's instructions.

As mentioned above, total RNA from individual animals was used for array hybridization (no pooling of samples) for the MAV, striatum, and parietal cortex. Five groups were evaluated, but only four individual animals could be analyzed per slide. To facilitate comparisons and reduce normalization variability, the first five of the 4 × 44 K slides groups were sequentially rotated so that 4 animals in all 5 groups were evaluated. The remaining EIH and AMPH animals needing evaluation were run on the next four slides, each of which contained one normothermic control and selected repeats of previously (first five slides) analyzed samples. A total of nine 4 × 44 K array slides for expression analysis were used for each region (27 slides total). This was done to aid in array normalization and to ensure that minimal variability occurred in expression results across all nine slides. There was minimal variability between duplicate control or treatment arrays, however, in our statistical analysis duplicates were not averaged because this could not be done for the AMPH groups. The same platform and very similar methods were followed to analyze the pools of RNA (two to three rats per pool) derived from the choroid plexus, except that the control pools were not run in duplicate. Five 4 × 44 K array slides were used for gene expression analysis of the choroid plexus.

Total RNA was reverse transcribed into complementary DNA (cDNA) using T7-promotor primer and MMLV reverse transcriptase. The cDNA was transcribed into complimentary RNA (cRNA), during which it was fluorescently labeled by incorporation of cyanine (Cy)3-CTP. After purification, using the RNeasy mini kit (Qiagen), cRNA yield and Cy3 incorporation efficiency (specific activity) into the cRNA were determined using a NanoDrop® ND-1000 Spectrophotometer (NanoDrop Technologies). All cRNAs had a yield >5 μg and a specific activity of 8–15 pmol/μg. For each sample, a total of 1.65 μg cRNA was fragmented and hybridized onto an Agilent 4 × 44 K oligonucleotide microarray in a hybridization oven at 65°C for 17 hr using the Agilent Gene Expression Hybridization Kit (5188–5281). The hybridized microarrays were disassembled at room temperature in Gene Expression Wash Buffer 1 (5188–5325) and then washed in Gene Expression Wash Buffer 1 at room temperature for 1 min. This was followed by a wash for 1 min in Gene Expression Wash Buffer 2 (5188–5326) at an elevated temperature (≈31°C) and air drying. To preserve the fluorescent signal intensity on the microarrays from degradation of the Cy3 dye due to environmental ozone, the entire disassembly and washing procedure was conducted in a low-ozone laboratory (~1 ppb ozone), with the final drying step and scanning conducted inside a low-ozone biobubble (<1 ppb ozone). The arrays were scanned on an Axon 4000B scanner (Molecular Devices Corporation, Sunnyvale, CA) and further processed using GenePix Pro 6.0 software (Molecular Devices). The resulting text files were uploaded into the ArrayTrack database (NCTR, Jefferson, AR) for analysis. The local background was subtracted from the fluorescence values of each spot to obtain signal intensity values.

The working microarray dataset was obtained through gene filtering, background correction, logbase2 (log2) transformation, and normalization. Probes were filtered to include only those genes with official NCBI gene symbols. We included in our analysis each probe for genes with less than 4 probes. For genes with 4 or more probes, we used the average signal intensity after discarding the minimum and maximum values. Raw expression data were corrected for background signal. To symmetrize the distribution of background-corrected intensity values and stabilize the variance, we transformed the gene expression data using log2 transformation and applied quantile normalization [66]. Statistical significance of differential expression was determined for all pairwise treatment comparisons within the 3 hr and 1 day time points. Significance was determined using the Significance Analysis of Microarrays (SAM) method [67]. SAM computes modified gene-specific t-test statistics and estimates the false discovery rate (FDR) using permutation analysis with replicated samples of the expression data. The FDR [68] is a statistical method for multiple hypothesis testing that controls the expected proportion of false positives among all identified genes in microarray analysis. Using SAM, we were able to identify differentially expressed genes while controlling the false discovery rate. We obtained each list of differentially expressed genes using SAM with an estimated FDR of approximately 10% for each pairwise comparison for 3 hr analysis and an FDR of approximately 20% for the 1 day analysis. Further, the lists of significant genes were filtered so that genes meeting the following criteria remained: at least one of the two genes in the pairwise comparison had an average intensity equal to or greater than 150 and the fold-change between the two treatments exceeded 1.3. We used the inverse of the log2 transformation to convert transformed/normalized data back to their original domain and used these values to report average gene intensity and fold-change.

Genes determined to be significantly different at the 3 hr and 1 day time points were analyzed using pathway analysis through Ingenuity Pathway Analysis (IPA) software (Ingenuity System, Inc., Redwood City, CA). The “Core Analysis” function in IPA was used to interpret the data in the context of biological processes, pathways, and networks.

### Quantitative RT-PCR analysis of gene expression

Aliquots of the total RNA used to generate first strand cDNA targets were diluted to 0.4 μg RNA/μl in RNase-free water for the reverse transcription (RT) reaction. The RT reaction was run using the standard techniques for use with Superscript™ III reverse transcriptase (Invitrogen Corp., Carlsbad, CA). Random hexamers (50 ng) were used for priming 0.8 μg of total RNA in a 10 μl reaction mixture that also contained 400 μM dNTPs. This mixture was incubated at 65°C for 5 min followed by 4°C for 5 min. Complementary DNA synthesis was then initiated by adding 10 μl of the reaction mixture to the priming mixture (20 μl total). The final concentrations of reactant constituents were 20 mM Tris–HCl (pH 8.4), 50 mM KCl, 5 mM MgCl_2_, 10 mM dithiothreitol, 200 μM dNTP, 2 units/μl RNaseOUT inhibitor, and 10 units/μl Superscript™ III reverse transcriptase polymerase. The reactants were first incubated for 10 min at 25°C, then 50°C for 50 min, followed by 85°C for 5 min. The mixture was then chilled on ice for 5 min, and 1 μl of RNase H was added to each sample followed by 20 min incubation at 37°C. The final cDNA product was diluted 20-fold and stored at −20°C until its use in polymerase chain reactions (PCR).

The expression of selected genes was determined by PCR, using iQ™ SYBR® Green Super mix (Bio-Rad Laboratories, Hercules, CA). The SYBR Green-labeled DNA products were amplified with 5’- and 3’-primers designed using the NCBI Primer-BLAST software and iQ™ SYBR® Green Super mix (Bio-Rad Laboratories, Hercules, CA), as previously described (Bowyer *et al.*, 2008). The Additional file 5: Table S5 lists the genes analyzed and the sequences of the oligonucleotide primers used. Each sample was run in duplicate. PCR cycling conditions were set at 95°C for 5 min for the first cycle and 30 seconds at 95°C followed by 30 sec at 60°C for the remaining 40 cycles. This was then followed by 40 repetitive cycles of 10 sec starting at 55°C and incrementing in temperature by 0.5°C/cycle to determine a melt curve as a means of validating the PCR product.

Relative quantities were calculated by subtracting the Ct values of the gene of interest from that of the endogenous control *Gapdh* (NM_017008) and expressed as a percentage of the *Gapdh* expression level by calculating 2^-ΔCt^ × 100. *Gapdh* Ct values that were used to normalize gene expression data can be found in the Additional file 5: Table S5. Genes with Ct values higher than 35 were considered not expressed (below the limit of quantification). For the 3 hr RT-PCR data, statistical significance between treatments for each gene was determined by carrying out the Kruskal-Wallis test followed by a multiple comparison of adjustment. The Mann–Whitney U test was used to analyze the difference between AMPH and EIH in the RT-PCR 1 day data. Statistical significance was evaluated at a 5% significance level (p < 0.05).

### Availability of supporting data

The data sets supporting the results of this article are included within the article and its four Supplementary Tables. The Oligo array data sets used for generating the Tables related to the array results for the choroid plexus can be found at GEO, NCBI; data file GSE29733, while those for the MAV, striatum, and parietal cortex can be found at GEO, NCBI; data file GSE23093.

## Abbreviations

AMPH: Amphetamine; BBB: Blood–brain barrier; CBF: Cerebroblood flow; CSF: Cerebrospinal fluid; EIH: Environmentally-induced hyperthermia; ERSR: Endoplasmic reticulum stress response; METH: Methamphetamine; MAV: Meninges and associated vasculature; NMDA: N-methyl-D-aspartate; ROS: Reactive oxygen species; RT-PCR: Quantitative real-time reverse transcription - polymerase chain reaction.

## Competing interests

The authors declare that they have no competing interests.

## Authors’ contributions

JB was involved in all aspects of this study with the exception of the actual generation of the oligonucleotide arrays. NG was involved in the interpretation and statistical analysis of the data and writing of the manuscript. TP was involved in technical assistance in generating the oligo arrays, in the interpretation and statistical analysis of the data, and writing of the manuscript. US was involved in technical assistance and generation of the oligo array data, in the interpretation and statistical analysis of the data, and writing of the manuscript. JH helped in experimental design, interpretation of the data and writing the paper. MT was involved in all aspects of this study except the RT-PCR analysis. LC generated the RT-PCR data and was involved in data analysis and interpretation and writing of the manuscript. JC was involved in the interpretation and statistical analysis of the data and writing of the manuscript. All authors read and approved the final manuscript.

## Supplementary Material

Additional file 1: Table S1 Normalized gene expression in control animals for all four brain regions retermined by the Agilent-014879 Whole Rat Genome 4x44K 60mer oligonucleotide arrays (G4131F, Agilent Technologies, Palo Alto, CA; NCBI GEO Accession # GPL7294). Click here for file

Additional file 2: Table S2 Putative function of genes with a 15-fold* or more increased expression in MAV compared to striatum and parietal cortex under control conditions. Click here for file

Additional file 3: Table S3 MAV genes significantly affected at 3 hr after EIH or AMPH exposure relative to control or relative to each other. Click here for file

Additional file 4: Table S4 Normalized choroid plexus expression of all genes at 3 hr after saline (control), AMPH or EIH. Click here for file

Additional file 5: Table S5 Sequences of the oligonucleotides used for the gene expression analysis by quantitative RT-PCR (FWD: forward; RV: reverse). Click here for file
