# Supplementary material for: Comparison of the global gene expression of choroid plexus and meninges and associated vasculature under control conditions and after pronounced hyperthermia or amphetamine toxicity
Source: BMC Genomics. 2013 Mar 5;14:147. doi: 10.1186/1471-2164-14-147 (PMC3602116; doi:10.1186/1471-2164-14-147)
Supplement: Additional file 2: Table S2 — Putative function of genes with a 15-fold* or more increased expression in MAV compared to striatum and parietal cortex under control conditions. [file 1471-2164-14-147-S2.doc]

**Supplementary Table 2 Putative function of genes with a 15-fold* or more increased expression in MAV compared to striatum and parietal cortex** under control conditions.

| MAV Expression/  Brain Expression | NCBI Gene Symbols | Tissue Specificity or Reported Function(s) |
| --- | --- | --- |
| > 50-fold | *Anxa2^a^, Col8a1, Des, Esm1, Glycam1, Lect1, Lepr, Lum, Myh11, Ogn, Tagln, Thbs2, Tnnt2* | Vasculature & Heart |
| > 50-fold | *Ccl19, Cd74^a^, Defb1, Lrrc21, Mgl1, Mrc1, Msln, Pla2g5, Prg4, RT1-Bb, RT1-Da* | Immune System |
| > 50-fold | *Adh1, Aebp1, Aldh1a2, Gstm2* | Retinoic Acid & Lipid Processing |
| > 50-fold | *Cdh1, Col3a1, Col1a2, Colec12, Cpxm2, Cpz, Dcn, Emp3, Gpc3, Kl^a^, Nupr1, Omd, Pcolce Slamf9, Tmem27, Tspan8* | Extracellular Matrix & cell-cell junctions |
| > 50-fold | *Aqp1, Asgr1, Kcnj13, Slc5a5, Slc6a13, Slc6a20, Slc22a6, Sned1, Ttr* | Ion & Solute Transport & Homeostasis |
| > 50-fold | *Alx3, Cdkn1c, Foxc2, Igfbp2, Ifitm1, Ifitm2, Mpzl2, Nkx6-1, Osr1, Prrx2, Sfrp1, Tbx15. Upk1b, Wisp2, Wnt6* | Development & Transcription Regulation |
| > 50-fold | *Gpha2, Mfap5, Plac8, Scgb1c1, Sostdc1, Steap1* | Unknown & Miscellaneous |
| 30 to 50-fold | *Anxa1^a^ , Angpt2, C6^a^, Gjb2, Ptgis, Thbd, Timp1* | Vasculature & Heart |
| 30 to 50-fold | *Casp12, Ccl2, Ccr1, Cd14, Cxcl10, Ifitm3, Klra5, Lgals1, Lgals3, Ms4a4a, Ms4a7, Plscr1, Spp1, Xcl1* | Immune System |
| 30 to 50-fold | *Col6a3, Cpxm1, Efemp1, Fmod, Mgp, Nid2* | Extracellular Matrix & cell-cell junctions |
| 30 to 50-fold | *Cp, Cubn, Gcgr, S100a6, Scn7a, Sct, Slc4a5, Slc16a11, Slco1a5* | Ion & Solute Transport & Homeostasis |
| 30 to 50-fold | *Cfd, Ch25h, Crabp2, Rarres2* | Retinoic Acid & Lipid Processing |
| 30 to 50-fold | *Bmp6, Casp12, Dab2, Folr1, Igf2, Msx1, Tbx18, Twist1, Wnt5b* | Development & Transcription Regulation |
| 30 to 50-fold | *Cela3b, Cln6, C1qtnf7, Copz2, Dhrs7c, Gng11, Prss23, Srpx, Vim* | Unknown & Miscellaneous |
| 15 to 30-fold | *Adm, Angpt1, Angptl2, Bgn, Cklf^a^, Cnn1,Cox8h, Ctsk, F13a1, Gja5, Klf4, Lox, Lyz, Myl9, Procr, Pros1, RT1-Ba, Serpinb10, Serping1, Serpinf1, Tgm2, Tnmd, Trim63, Txnip^a^, Vamp5, Vtn* | Vasculature & Heart |
| 15 to 30-fold | *Ada^a^, Bst2, Ccl6, Cd40, Cd68, Ctsc, Dap, Faim3, Fkbp9, Fxyd5, Fmo1, Glipr1, Ier3, Ifi47, Igsf6, Msc, Nfatc4, RT1-Db1, Serpinb1a, Tir4, Tubb6* | Immune System |
| 15 to 30-fold | *Adamtsl4, Col1a1, Crb3, Dpt, Egfl3, Fbn1, Fbln1, Fbln5, Fn1, Itgb4, Lama2, Lgals3bp, Loxl1, Mfap4, Mmp14, Mmp23, Ppic, Serpinh1, Timp3* | Extracellular Matrix & cell-cell junctions |
| 15 to 30-fold | *Cybrd1, Selenbp1, Slc2a4, Slc9a2, Slc13a3, Slc16a4, Slc22a8, Slc22a18* | Ion & Solute Transport & Homeostasis |
| 15 to 30-fold | *Agpat2, Bdh2, Cyp26b1, Lpar3, Ltb4dh, Olr1, Pon3, Rbp1, Rbp4* | Retinoic Acid & Lipid Processing |
| 15 to 30-fold | *Atf3, Dkk4, Eya2, Ifitm7, Ltbp1, Mustn1, Nr2f2, Ptrf, Sphk1, Tgfbi, Tcea3 Tcfap2b, Wnt2b, Wnt5a, Zic1, Zic4* | Development & Transcription Regulation |
| 15 to 30-fold | *Adamts12, Adamtsl3, C1r, Crispld2, Crygn, Cyp1b1, Dse, Enpep, Enpp2, Epn3, Flna, Fmo3, Gnmt, Gprc5c, Hspb1, Klc3, Krt18, Krt19, Mdk, Mesp1, Ms4a6a, Ms4a6b, Ms4a11, Net1, Pdlim2, Phactr2, Plp2, Ppp1r3b, Pqlc3, Rin3, Spag11, Sult1a1, Tmem106a, Wfikkn2Top of Form* | Unknown & Miscellaneous |

* Genes included in the table must have been more than 15-fold above expression in the MAV compared to both the striatum and parietal cortex and at least 5-fold above the background level. Also, expression must have been relatively consistent across individual control animals so that the S.D. was less than the mean. The lower of the two ratios (MAV/striatum or MAV/parietal cortex) for each gene was used for grouping. Genes were categorized using *NCBI Entrez Gene.* Note: a table similar to this was previously shown in [41] but without references or comparisons to choroid plexus data which has not been previously presented.

^a^ Genes found in endothelial cells but also likely play a major role in mediating immune responses.

Genes in red font indicate they were also at least 15-fold or more in the choroid plexus compared to both the striatum and parietal cortex.
